# Dynamics of *Panax ginseng* Rhizospheric Soil Microbial Community and Their Metabolic Function

**DOI:** 10.1155/2014/160373

**Published:** 2014-08-19

**Authors:** Yong Li, YiXin Ying, WanLong Ding

**Affiliations:** Institute of Medicinal Plant Development, Chinese Academy of Medical Sciences and Peking Union Medical College, No. 151 Malianwa North Road, Beijing 100193, China

## Abstract

The bacterial communities of 1- to 6-year ginseng rhizosphere soils were characterized by culture-independent approaches, random amplified polymorphic DNA (RAPD), and amplified ribosomal DNA restriction analysis (ARDRA). Culture-dependent method (Biolog) was used to investigate the metabolic function variance of microbe living in rhizosphere soil. Results showed that significant genetic and metabolic function variance were detected among soils, and, with the increasing of cultivating years, genetic diversity of bacterial communities in ginseng rhizosphere soil tended to be decreased. Also we found that *Verrucomicrobia*, *Acidobacteria*, and *Proteobacteria* were the dominants in rhizosphere soils, but, with the increasing of cultivating years, plant disease prevention or plant growth promoting bacteria, such as *Pseudomonas*, *Burkholderia*, and *Bacillus*, tended to be rare.

## 1. Introduction

Ginseng (*Panax ginseng* C.A. Meyer) is mainly cultivated in China, Korea, and Japan. It has been regarded as one of the most important remedies in oriental medicine for more than 1,000 years [1]. Nowadays, it is usually used as adaptogenic, antiaging health tonic, and so forth. As herbaceous perennial plant, ginseng requires at least 6 years of growth from sow to harvest. In China, after growing 2 or 3 years, ginseng usually is transplanted to another site until harvest. During the long growing process, soilborne diseases made a severe threat on the health of* P. ginseng* root.

Rhizosphere soil is defined as soil tightly adhering with plant root [2]. Plant releases a series of compounds through root into rhizosphere soil which provide plentiful nutrition to rhizosphere microbe [3]. On the other hand, rhizosphere bacteria play an important role in nutrient cycling, organic matter decomposition, and soil fertility maintaining [4]. Recently, though a few novel bacterial strains have been isolated from field plant ginseng ever [5–7] or from the interior of ginseng root [8, 9], most of the bacterial community in ginseng rhizosphere soil is still unknown yet.

In the present study, culture-independent methods, random amplified polymorphic DNA (RAPD), and amplified ribosomal DNA restriction analysis (ARDRA) were used to examine the bacterial community and dynamics of dominant bacterial species in ginseng rhizosphere soil during the growth of* P. ginseng*. Furthermore, Biolog EcoPlate was used to study the metabolic function variance of rhizosphere microbe. The aim of the present study was to reveal the dynamics of rhizosphere bacterial communities during the growth of* P. ginseng* by culture-dependent and culture-independent methods.

## 2. Experimental Section

### 2.1. Soil Collection and DNA Extraction

Rhizosphere soils of one- to six-year ginseng were sampled from Dafang (H: 570.8 m N: 42°26′03.2′′ E: 127°20′00.1′′) in Fusong county, Jilin Province, China, in January 2009. The soil is uniform with histosols soil according to the UN-FAO soil classification system. For each sample, soil tightly adhering on the surface of five healthy ginseng roots at the same field was collected. The genomic DNA of soil microbes was extracted immediately.

Genomic DNA was extracted from 0.5 g fresh soil using E.Z.N.A. Soil DNA Kit (OMEGA, USA) according to the manufacturer's instructions. The successful extraction of genomic DNA was checked by 0.8% agarose gel electrophoresis with 1 × TAE buffer (2 mol/L Tris-base, 50 mmol/L EDTA, and 1 mol/L acetic acid, pH 8.0).

### 2.2. RAPD Fingerprinting

Genetic diversity of microbes in ginseng rhizosphere soils was examined by RAPD method. Amplification was performed in a 25 *μ*L volume containing 20 ng template DNA, 0.2 *μ*mol/L primer, 100 *μ*mol/L dNTP, 1 × PCR buffer (10 mmol/L Tris-HCl, pH 8.0, 50 mmol/L KCl, 1.5 mmol/L MgCl_2_), and 1 U Taq DNA polymerase. In total, 15 repetitive and polymorphic primers (OPH11, OPI4, OPJ1, OPJ4, OPJ7, OPR7, OPR8, OPR10, OPR11, OPR14, OPR17, OPS4, OPS10, OPT16, and OPT17) were used for RAPD analysis. Amplification was performed in a T Gradient 96 Thermal Cycler (Biometra) with cycling program that consisted of initial denaturation of 1 min at 94°C, followed by 40 cycles of 1 min denaturation at 94°C, 1 min annealing at 37°C, 1.5 min extension at 72°C, and a final extension at 72°C for 7 min.

Products amplified were separated on 1.2% agarose gels containing ethidium bromide, and reproducible, clear bands from 100 bp to 1500 bp were recorded. Fingerprinting profile was then converted to a two-dimensional binary matrix (“1” indicates presence of band; “0” indicates absence of band) and calculated using NTSYSpc version 2.10e software for clustering analysis [10]. The dendrogram was constructed using the unweighted pair group method (UPGMA). Nei's genetic diversity and Shannon's information index were calculated by population genetic analysis software Popgene version 1.32 (32-bit).

### 2.3. Amplified Ribosomal DNA Restriction Analysis (ARDRA)

The bacterial community was analyzed by a cultivation-independent method. 16S ribosomal DNA of bacteria was amplified by a pair of universal primers 27f (5′-AGA GTT TGA TCM TGG CTC AG-3′) and 1492r (5′-TAC GGY TAC CTT GTT ACG ACT T-3′) [11, 12] in a T Gradient 96 Thermal Cycler. The successful amplification was checked by 1% agarose gel electrophoresis.

Target fragments were purified by Wizard PCR Preps DNA Purification System (Promega, USA), ligated with PMD-18T vector (TaKaRa), and then transferred into* E. coli* TOP10 competent cell according to the manufacturer's instructions. Aliquot (100 *μ*L) of each transformation was spread on LB/ampicillin/IPTG/X-gal plates and incubated at 37°C for 16 h. For each sample, 192 white colonies were picked out, which were amplified by 27f and 1492r primer that the positive clones were confirmed through. Then, 10 *μ*L target insert fragments (about 1,500 bp) were digested by 3 U restriction endonuclease Hinf I (TaKaRa) [13, 14] and Pst I (TaKaRa) [15] at 37°C for 2 h. Clones having the same restriction patterns were defined as an operational taxonomic unit (OTU).

Representative clones of unique ARDRA patterns were sequenced by automated DNA capillary sequencer 3730 (Applied Biosystems, USA). The partial sequences of 16S rRNA gene were blasted with known 16S rDNA sequences in GenBank databases using nucleotide BLAST program (http://blast.ncbi.nlm.nih.gov/blast.cgi) [16]. The saturation of clones in the library was evaluated by rarefaction curves [17] calculated using the Analytic RarefactWin Version 1.3 (http://www.uga.edu/~strata/software/index.html) program [18].

### 2.4. Metabolic Characteristics of Soil Microbe

Soil metabolisms of soil microbial communities were characterized by community level physiological profiles (CLPP) using Biolog EcoPlate [19]. Ten grams of fresh soil was suspended in 90 mL of sterile 0.85% saline solution and shaken at 120 rpm for 30 min, and then suspensions were diluted 1,000-fold. Each well of a Biolog EcoPlate was inoculated with 150 *μ*L diluents and incubated at 25°C in dark without agitation. The plates were scanned at wavelength of 590 nm by a Biolog reader on OmniLog Plus (BIOLOG Inc., Hayward, CA, USA) at a 24-hour interval for 168 h. Each soil sample using one plate with 31 carbon substrates is arranged in triplicate.

The average well color development (AWCD) was used to evaluate the general carbon substrates utilization ability [9, 20], where “*A*_*i*_” is the absorption of *i*th well and “*A*_*A*_1__” is the absorption of the “*A*_1_” well following the incubation measured in terms of the optical density at wavelength of 590 nm (OD590). AWCD of each well was calculated using the following formula:

(1) $$\begin{array}{c} \text{AWCD}=\frac{\sum \left(A_{i}-A_{A_{1}}\right)}{31}. \end{array}$$

The metabolic profile of microbial community includes the Shannon index (*H*′) and the evenness index (*E*) [21, 22]. The diversity of microbial community was evaluated by the Shannon index (*H*′) [23], calculated using the formula

(2) $$\begin{array}{c} H^{\prime }=-\overset{s}{\underset{i=1}{\sum }}p_{i}\cdot \ln p_{i}, \end{array}$$

where “*p*_*i*_” is the principal color development of the “*i*th” well relative to the total color development, that is, *p*_*i*_ = (*C* − *R*)/∑(*C* − *R*), and “*s*” is the summation of absorption values of all wells in a Biolog EcoPlate. The evenness index was calculated using the formula *E* = *H*′/ln⁡ *S*, where diversity “*S*” is the total number of carbon substrates utilized by microbial community in a given soil sample, and only the positive data, the optical density (OD) ≥0.2, was used to calculate “*S*.” The AWCD value at 120 h was used to calculate the Shannon index (*H*′); SPSS 17.0 and SIMCA-P 11.5 Demo software were used for PCA analysis [24].

## 3. Results

### 3.1. RAPD Analysis

Bacterial diversity indices *H*′ and *I* decreased in soil B compared to soil A but increased sharply in soil C. *H*′ and *I* of soils D, E, and F were significantly lower than of soils A, B, and C. Soil D has the lowest indices, while soil C has the highest indices (Table 1).

**Figure 1:**
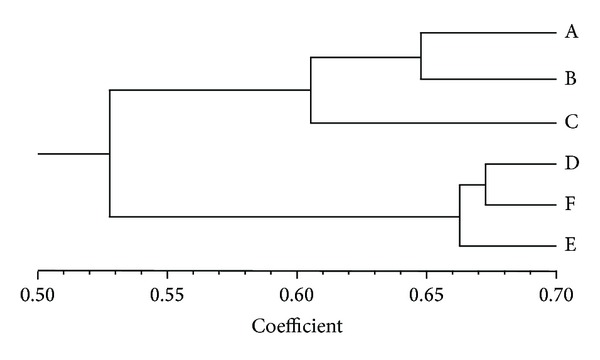
UPGMA dendrogram of six ginseng rhizosphere soils.

Clustering results showed that, under the 0.58 coefficient threshold, 6 soil samples were divided into two groups. Group I included soils A, B, and C, while group II included soils D, E, and F. The highest similarity coefficient was detected between soils D and F (Figure 1).

### 3.2. ARDRA and Phylogenetic Analysis

In total, 167 OTUs were generated from 961 clones, in which 27, 27, 44, 16, 28, and 25 OTUs were identified in soils A, B, C, D, E, and F, respectively.

**Figure 2:**
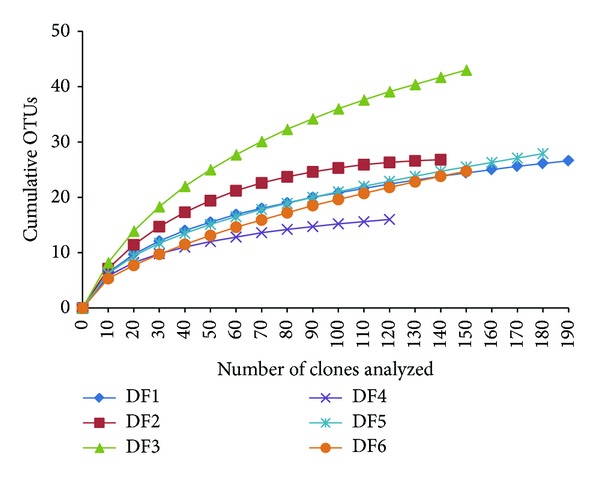
Rarefaction curves for bacterial OTUs, clustering at 97% rRNA gene similarity.

The saturation of OTUs analyzed was evaluated by rarefaction curves, which indicated that six clone libraries were near the saturated status (Figure 2). ARDRA analyzing results indicated that soil D has the lowest diversity (16 OTUs), whereas diversities of soil C (44 OTUs) were the highest.

**Figure 3:**
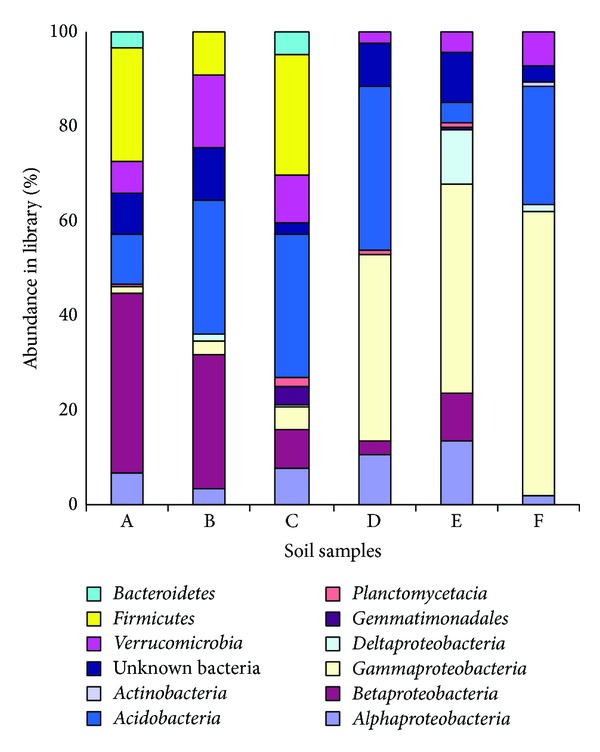
Bacterial communities in ginseng rhizosphere soil.

Sequencing results indicated that* Verrucomicrobia*,* Acidobacteria*, and* Proteobacteria* were the dominants in six soils. Also,* Gemmatimonadetes*,* Planctomycetes*,* Firmicutes*,* Bacteroidetes*,* Actinobacteria*,* Gemmatimonadales*, and unclassified bacteria were identified in soils.* Proteobacteria* showed the most significant differences among 6 soils.* Firmicutes* are only present in soils A, B, and C.* Gemmatimonadales* are only present in soils C and E.* Bacteroidetes* are only present in soils A and C. *α*- and *γ*-*Proteobacteria* are present in 6 soils simultaneously. *γ*-*Proteobacteria* constitute a substantial proportion of clones (about 60%) in soil F. Except for soil F, *β*-*Proteobacteria* are present in another 5 soils. *δ*-*Proteobacteria* are present in soils except soils A and E.* Verrucomicrobia* are another major group present in 6 soils.* Actinobacteria* are present in soil F. Except for soils B and F,* Planctomycetes* are present in another 4 soils with a small proportion. Unclassified bacteria (about 8.5% of total) are detected in 6 soils. Of the 167 OTUs sequenced, 63.2% (607 clones) had higher similarity to the 16S rDNA sequences of uncultured bacteria, and only 36.8% (354 clones) were most closely related to cultured isolates (Figure 3).

### 3.3. BIOLOG Analysis

**Figure 4:**
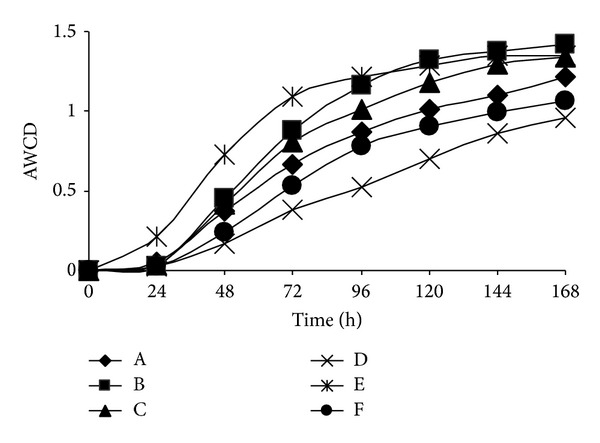
Average well color development (AWCD) with incubation.

As a universal indicator of metabolic activity, AWCD changes were shown in Figure 4. Obviously, the metabolic activity tended to be increasing along with incubation time. However, metabolic activity among soils showed significant differences. For example, soils D and F usually had the lowest AWCD, which indicated that their metabolic activity was the lowest.

According to the curve of AWCD versus the culturing time, the AWCD values in 120 h were used to describe the difference of soil microbial metabolic activity. In the present research, the order of metabolic activity based on AWCD was described as follows: soil D < soil F < soil A < soil C < soil E < soil B. The Shannon diversity ranged from 2.664 to 2.791; soil F has the lowest Shannon diversity and evenness indices (Table 1).

**Figure 5:**
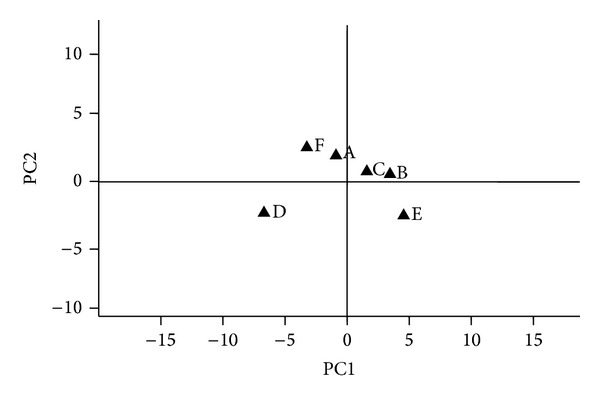
Principal component analysis (PCA) of Biolog EcoPlates data. Each solid triangle represents a soil sample.

To show which types of the substrates were utilized and the intrinsic differences between microbial communities, principal component analysis was then performed to display the variance of microbial communities (Figure 5), which could clearly separate the soil samples according to the different age of ginseng. The first principal component (PC1) and the second principal component (PC2) contributed 58% and 18% to the total variation, respectively. By substrate utilization patterns, soil samples were clearly divided into two groups: one with soil samples D, F, and A and the other with soils C, B, and E. The difference of C utilization patterns supported the fact that ginseng of different ages had significant influence on rhizosphere soil microbial community (Table 2).

The substrates with high correlation coefficients to PC1 and PC2 were shown in Table 2. It is illustrated that carboxylic acids and carbohydrates influence PC1 greatly, which were carbohydrates and amino acids for PC2.

## 4. Discussion

Culture-independent method was used to investigate the bacterial community; the dominant bacteria in ginseng rhizosphere soils were not the same as those found from a wide range of soils, such as pristine forest, grassland, and agricultural soils [25]. Such differences could likely be explained by different soil characteristics [26]. Root exudates released by plant provide plenteous nutrition for rhizospheric microorganisms and had a great influence on the microbial community. So we deduced that ginseng is a herbaceous perennial plant growing at a special environment, and its exudates are different from others, which finally resulted in special bacterial community.

According to RAPD and ARDRA analysis, soil C has the highest diversity index. It was also found that bacterial diversity in rhizosphere soil of cotton increased from squaring period to flowering period [27]. The reason for this could be that young roots are known to excrete more organic material than older roots, which can result in different specific bacterial populations [28]. Among soils tested, soils D and F have the lowest diversity index. Further analysis indicated that carbon sources metabolic activity of two soils were also the lowest. Although the relationship between biological diversity and ecological function of soil bacterial community has not been fully understood, the decrease of microbial diversity will obviously result in the loss of some ecological function and finally make the ecological system unhealthy [29–31]. Actually, soils ever cultivated ginseng, which are traditionally called “old ginseng soil,” such as soils D and F, which are not suitable for the growth of the next generation of ginseng. So we deduced that the decreased genetic diversity and reduced ecological function disorder made the soil unsuitable for ginseng growth further.

Rhizosphere is a unique environment, where pathogens and beneficial microbe have important influence on the growth and health of plants [32].* Pseudomonas* and* Burkholderia* belong to* Proteobacteria*, and* Bacillus* belongs to* Firmicutes* which were reported to have antagonistic activity against soilborne pathogenic fungi, such as* Rhizoctonia*,* Sclerotinia*,* Verticillium,* and* Gaeumannomyces* [33–35]. In the present study, genera of* Pseudomonas*,* Burkholderia*, and* Bacillus* were found in soils A, B, C, and E. Actually, during the cultivation, 6-year-old ginseng is more easily infected by soilborne pathogenic fungi. So we deduced that the decrease of the* Pseudomonas*,* Burkholderia*, and* Bacillus* in rhizosphere soil may be a key cause that resulted in 6-year ginsengs being more easily infected by soilborne pathogens.

It is known that soil bacterial populations are influenced by a wide range of factors. Soil type, plant species, and cropping patterns are the factors that most affect the bacterial community structure in soil [8, 36]. In order to reduce interference from other factors and truly reflect the relationship between soil bacterial succession and continuous cropping with ginseng plants, several measures were used, including the uniformity of management of ginseng cultivation. Cluster analyses demonstrated that the soil bacterial assemblages obtained from the same cropping cycle were similar; genetic polymorphic analyses and carbon metabolic analyses also showed dynamic changes in bacterial populations with continuous ginseng cropping. It has been reported that soil microbial biomass and their structure were also significantly influenced by continuous cropping with the other grain crops or economic crops [37, 38]. These findings indicated that successional change in soil microbial communities with continuous cropping may be a common feature.

## Figures and Tables

**Table 1:** Bacterial diversity and metabolic function indices of ginseng rhizosphere soils.

Soil samples	Cultivating year	*H*′	*I*	Shannon diversity	Evenness
A	1	0.4782	0.6712	2.791	0.829
B	2	0.4357	0.6273	2.786	0.827
C	3	0.4880	0.6811	2.768	0.838
D	4	0.3519	0.5367	2.716	0.832
E	5	0.3866	0.5750	2.778	0.822
F	6	0.4142	0.6047	2.664	0.818

*H*′ indicates Nei's gene diversity; *I* indicates Shannon's information index.

**Table 2:** Substrates highly correlated with PC1 and PC2.

PC1	*r*
Carbohydrates	
I-Erythritol	0.895
Glycogen	0.835
D-Glucosaminic acid	0.859
D-Cellobiose	0.891
Amino acids	
L-Arginine	0.848
L-Phenylalanine	0.918
L-Threonine	0.838
*Carboxylic acids *	
Pyruvic acid methyl ester	0.873
y-Hydroxybutyric	0.930
Itaconic acid	0.959
a-Ketobutyric acid	0.907
Amines	
Putrescine	0.968
Phenolic	
2-Hydroxybenzoic acid	0.802
4-Hydroxybenzoic acid	0.905
Polymer	
a-Cyclodextrin	0.886

PC2	*r*

Amino acids	
L-Asparagine	0.805
Carbohydrates	
N-Acetyl-D-glucosamine	0.804
